# Histo-Blood Group Gene Polymorphisms as Potential Genetic Modifiers of Infection and Cystic Fibrosis Lung Disease Severity

**DOI:** 10.1371/journal.pone.0004270

**Published:** 2009-01-26

**Authors:** Jennifer L. Taylor-Cousar, Maimoona A. Zariwala, Lauranell H. Burch, Rhonda G. Pace, Mitchell L. Drumm, Hollin Calloway, Haiying Fan, Brent W. Weston, Fred A. Wright, Michael R. Knowles, for the Gene Modifier Study Group

**Affiliations:** 1 University of New Mexico Health Sciences Center, Pulmonary Divisions, Internal Medicine and Pediatrics, Albuquerque, New Mexico, United States of America; 2 University of North Carolina, Cystic Fibrosis/Pulmonary Research and Treatment Center, Chapel Hill, North Carolina, United States of America; 3 National Institute of Environmental Health Sciences, Research Triangle Park, North Carolina, United States of America; 4 University of North Carolina, Department of Biostatistics, Chapel Hill, North Carolina, United States of America; 5 University of North Carolina, Hematology-Oncology, Department of Pediatrics, Chapel Hill, North Carolina, United States of America; 6 Case Western Reserve University, Pediatric Pulmonary Division, Department of Pediatrics, Cleveland, Ohio, United States of America; Ohio State University Medical Center, United States of America

## Abstract

**Background:**

The pulmonary phenotype in cystic fibrosis (CF) is variable; thus, environmental and genetic factors likely contribute to clinical heterogeneity. We hypothesized that genetically determined ABO histo-blood group antigen (ABH) differences in glycosylation may lead to differences in microbial binding by airway mucus, and thus predispose to early lung infection and more severe lung disease in a subset of patients with CF.

**Methods and Principal Findings:**

Clinical information and DNA was collected on >800 patients with the ΔF508/ΔF508 genotype. Patients in the most severe and mildest quartiles for lung phenotype were enrolled. Blood samples underwent lymphocyte transformation and DNA extraction using standard methods. PCR and sequencing were performed using standard techniques to identify the 9 SNPs required to determine ABO blood type, and to identify the four SNPs that account for 90–95% of Lewis status in Caucasians. Allele identification of the one nonsynonymous SNP in FUT2 that accounts for >95% of the incidence of nonsecretor phenotype in Caucasians was completed using an ABI Taqman assay. The overall prevalence of ABO types, and of FUT2 (secretor) and FUT 3 (Lewis) alleles was consistent with that found in the Caucasian population. There was no difference in distribution of ABH type in the severe versus mild patients, or the age of onset of *Pseudomonas aeruginosa* infection in the severe or mild groups. Multivariate analyses of other clinical phenotypes, including gender, asthma, and meconium ileus demonstrated no differences between groups based on ABH type.

**Conclusions and Significance:**

Polymorphisms in the genes encoding ABO blood type, secretor or Lewis genotypes were not shown to associate with severity of CF lung disease, or age of onset of *P. aeruginosa* infection, nor was there any association with other clinical phenotypes in a group of 808 patients homozygous for the ΔF508 mutation.

## Introduction

Cystic fibrosis (CF) is the most common life shortening genetic disorder in Caucasians, and occurs in 1 in 3200 Caucasians in the U.S. [1] Since the identification of the CF gene in 1989 [2], over 1500 mutations in the Cystic Fibrosis Transmembrane Conductance Regulator (CFTR) have been associated with CF. [3] The most common mutation, ΔF508, occurs in ∼70% of CFTR alleles. [1] Although numerous mutations in the CF gene have been described, there is little correlation between the specific mutation in the CF gene and severity of pulmonary disease. Recent investigations have focused on the identification of other non-CF gene variants that may affect the phenotype, i.e. “modifier genes.” [4], [5]

Mutations in CFTR cause CF, and the initial pathophysiology of the lung disease involves defective ion transport, dehydration of the mucous layer, and reduced mucociliary and cough clearance. [6] These abnormalities lead to chronic bacterial infection, which is the key factor in the morbidity and mortality of CF lung disease [7], [8]; additionally, respiratory viral infections are associated with exacerbation and progression of lung disease. [9], [10]

Bacteria and viruses bind to mucins and the cellular glycocalyx of airway epithelia based on their glycosylation patterns[11]; thus, genetically based differences in patterns of glycosylation and microbial binding may increase the likelihood of airway infection, and contribute to progression of airway damage, and severity of lung disease. One cause of variability in glycosylation of airway epithelia is inherited blood type, which reflects genetic-based differences in glycosylation enzymes. The histo-blood group antigens, ABO, H, and Lewis, are formed by the action of glycosyltransferases to attach sugar molecules to disaccharide precursors. (See Supplement Text S1 and Figure S1.) Addition of fucose to these disaccharide precursors creates the H antigen, and further modification to the H antigen by glycosyltransferases leads to synthesis of the blood group antigens encoded by the ABO (“blood type”) gene. The O allele does not produce an active enzyme. [12]

Formation of other histo-blood group antigens is dependent on fucosyltransferases. The FUT2 “secretor” gene encodes the fucosyltransferase that synthesizes the H antigen. Loss-of-function mutations in FUT2 (secretor gene) lead to an absence of ABH antigens in the lung, saliva, and gut, resulting in the “non-secretor” phenotype. Loss-of-function mutations in the FUT3 (Lewis) gene result in the Lewis negative phenotype. [11]

The ABO gene is highly polymorphic, which is thought to be related to evolutionary changes for host-pathogen interactions, as many pathogens utilize surface glycoproteins in host invasion. [11] There is correlation with ABH status and infection with bacteria and viruses; for example, numerous studies have shown that the binding and infection of humans with noroviruses (previously Norwalk virus,) is dependent on ABO and secretor phenotype. [13], [14] Furthermore, certain strains of *S. aureus* bind to the Lewis antigen [15], and *P. aeruginosa* has a lectin, PA-IIL, that is fucose-specific. [16] Thus, genetic-based differences in glycosylation that determine blood type can also predispose to an increased likelihood of bacterial and/or viral binding and infection in the airways. Additionally, polymorphisms in the genes coding for the ABH antigens are associated with airway diseases, including COPD and asthma. [17], [18], [19]

We speculated that genetic predisposition to different patterns of glycosylation in the ABH pathway would lead to early chronic lung infection in CF, and therefore, more severe progression of disease. We tested the genotype of key (ABH) glycosylation enzymes in CF patients with “severe” versus “mild” lung disease, and tested for associations with age of onset of infection with *P. aeruginosa.*

## Methods

### Objectives

1. Establish ABO, FUT2 and FUT3 genotype for each subject
2. Determine whether differences in ABH type predict differences in lung phenotype.

We hypothesized that subjects with ABH types with more glycosylation (i.e. blood groups A, and B, and secretor and Lewis positive phenotypes) would be more likely to have severe lung phenotype as a result of having an increased predisposition to early viral/bacterial infection.

### Participants

Patients with Cystic Fibrosis homozygous for the ΔF508 CF gene mutation were enrolled from 44 sites (n = 808 patients) as previously published. [20] Patients were enrolled based on FEV_1_ values in the lowest quartile (“severe;” n = 263) or highest quartile (“mild;” n = 545) for age. FEV_1_ data has been analyzed according to biostatistical models based on longitudinal lung function (FEV_1._) This modeling has robust power to predict survival; hence the use of multiple spirometric values is a strong surrogate for long-term outcomes. [21]

### Description of Procedures

#### Data Collection

Each patient received a unique identifier code to maintain confidentiality. Source documents were collected for pulmonary function tests, meconium ileus history and sputum microbiology. Case report forms were used to collect information about the presence of diabetes mellitus, steroid use, oral hypoglycemic and insulin use.

#### Source of DNA

Whole blood in sodium citrate (for DNA extraction) and heparinized (for lymphocyte transformation) tubes was collected from all patients at the time of study enrollment. DNA was extracted and stored using standard methodology in the central molecular pathology facility at University of North Carolina-Chapel Hill (UNC) Hospitals. DNA and transformed lymphocytes were stored frozen in two different storage containers at two separate sites with continuous monitoring.

#### ABO Genotyping

Sequence variation in 7 ABO alleles accounts for >90% of the variants in white Europeans. [12] Primers were designed to amplify three fragments from exons 6 and 7 containing the 9 SNPs (rs8176719, rs8176720, rs1053878, rs7853989, rs8176740, rs8176741, rs8176742, rs816750, rs8176472) necessary to identify blood type. (See Supplement Text S1 and Table S1 for primer sequences.) PCR was performed using standard techniques, and high throughput sequencing was performed on an ABI Prism® 3730 Genetic Analyzer. Batch data analysis was performed with SNP finding via the Polyphred© program. (See Supplement Text S1 and Table S2 for sequence variation information used to construct ABO phenotype.)

#### FUT2 Genotyping

Loss of function mutations in FUT2 (non-secretor) occur in 20% of European-derived Caucasians. In Caucasians, one nonsynonymous SNP in FUT2 (G428A, rs601338) accounts for >95% of the incidence of nonsecretor phenotype. [22] Allele identification was performed using an ABI Taqman© assay on an ABI Prism® 7700HT/7900HT Sequence Detection System.

#### FUT3 Genotyping

Loss of function mutations in FUT3 (Lewis negative) occur in 10% of European-derived Caucasians. SNPs in four positions (T59G, rs28362459; T202C, rs812936; C314T, rs778986; and T1067A, rs3894326) account for 90–95% of Lewis status in Caucasians. [23] Primers were designed for PCR amplification, and sequencing of the FUT3 gene was performed on an ABI Prism® 3130xl/310 Genetic Analyzer. [24], [25]

#### Acquisition of *P. aeruginosa*

In addition to lung function data, all available CFF registry data on study subjects that related to respiratory tract (oropharyngeal and sputum) cultures was acquired. Age of onset of persistent *P. aeruginosa* infection for all patients was established with available culture data (n = 674), and defined as the first age at which *P. aeruginosa* was present in respiratory cultures in two consecutive, or two out of three years.

#### Ethics

This study was approved by Institutional Review Boards at all participating institutions. Each participant provided written informed consent prior to study participation. The study is registered in the ClinicalTrials.gov database: NCT00037765.

#### Statistical Methods

To test for associations between severity of lung disease, key clinical phenotypes, and polymorphisms in ABH genotypes, Fisher's exact test was used. All tests were two-sided. To investigate the effect of blood group alleles (ABO genotype, nonsecretor phenotype, and FUT3 genotypes producing Lewis negative phenotypes) on CF lung severity risk, we performed multiple logistic regression analyses to predict severity status. The number of A and B antigen alleles was included in an additive model on the log-odds of severe lung status, as well as the FUT2 and FUT3 genotypes, and terms to account for possible interaction between the genes. In addition, the following predictors were used in the model: gender, meconium ileus, asthma, and the TGFb1 codon 10 CC genotype (previously shown to be associated with severe lung disease.) In order to determine whether ABH type influenced the age of acquisition of *P. aeruginosa*, Kaplan-Meier analysis was performed using SigmaStat® software. ABO type, and secretor and Lewis status were evaluated in the mild versus severe groups. Additionally, to address the possibility of age confounding the analysis of *P. aeruginosa* acquisition, ABH types were also analyzed in the severe group versus the group of mild patients who were aged 15–28 years (“young milds,”) and interactions among the three ABH types were analyzed. Allelic comparison that yielded a statistical significance of ≤0.05 was considered significant. Unadjusted p-values are reported.

## Results

### Characteristics of the patients

As previously reported, pulmonary and nutrition characteristics of the patients classified as severe (n = 263) were markedly different than that of patients classified as mild (n = 545.) [20] Although the mean age of the patients in the mild group was 28.6 ± 9.7 years compared to 16.2 ± 4.2 years in the severe group, FEV_1_ was more than 25% higher and body mass index percentile was 24% higher in the patients in the mild group versus those in the severe group (p<0.001). Gender, prevalence of diabetes mellitus (adjusted for age), and asthma were similar between patients in the mild and severe groups. [20]

### ABO genotype

ABO genotype was successfully determined for 778 patients. ABO allele frequencies, and resultant ABO blood groups were consistent with the reported prevalence in the Caucasian population. [26], [27] (See Supplement Text S1 and Table S3.) We saw no significant difference in blood type distribution between mild and severe patients by Fisher exact test. (p = 0.446). (Table 1) We also saw no significant associations when ABO allele distribution was analyzed in the following subgroups: males and females, patients with and without asthma, and patients with and without history of meconium ileus. (Data not shown.)

**Table 1:** Prevalence of ABO blood types in severe and mild patient groups.

Blood type	Severe Group	Mild Group
**O**	127 (50.8%)	243 (46.2%)
**B**	21 (8.3%)	62 (11.8%)
**A**	94 (37.3%)	202 (38.4%)
**AB**	9 (3.6%)	19 (3.6%)
	252	526

ABO allele frequencies and resultant ABO phenotypes were consistent with known prevalence in the Caucasian population. [26] There is no significant difference in blood type distribution amongst mild and severe patients, or in any subgroup analysis (see Text S1) by Fisher exact test. (n = 778, p = 0.446)

### FUT2 genotype

FUT2 (“secretor/nonsecretor”) alleles for SNP G428A (rs601338) were successfully determined in 806 patients. The distribution of alleles was consistent with that found in the Caucasian population. [27] There was no significant difference in secretor status distribution between mild and severe patients by Fisher exact test (p = 0.569). (Table 2) Subgroup analysis by gender, asthma and meconium ileus status did not reveal any associations. (Table 2)

**Table 2:** Prevalence of FUT2 and FUT3 alleles and genotypes in severe and mild patient groups.

Gene	Variant	Reference SNP	Impairment of lung function	Genotype	Patients with genotype	Genotype	Patients with genotype	Genotype	Patients with genotype	Number of Patients	P-Value§
FUT2 (Secretor gene)	G428A	rs601338	Mild	AA	137(24.3%)	AG	273 (54.0%)	GG	134 (21.6%)	544	0.569
			Severe	AA	57 (21.8%)	AG	137 (52.3%)	GG	68 (26.0%)	262	
FUT3 (Lewis gene)	T59G	rs28362459	Mild	TT	389 (84.0%)	TG	71 (15.6%)	GG	3 (1.2%)	463	0.554
			Severe	TT	208 (82.2%)	TG	43 (16.9%)	GG	3 (1.2%)	254	
	T202C	rs812936	Mild	TT	318 (66.1%)	TC	127 (30.3%)	CC	18 (3.47%)	463	0.491
			Severe	TT	168 (66.1%)	TC	79 (31.1%)	CC	7 (2.8%)	254	
	C314T	rs778986	Mild	CC	321 (66.5%)	CT	126 (30.4%)	TT	16 (3.1%)	463	0.615
			Severe	CC	172 (67.7%)	CT	76 (29.9%)	TT	6 (2.4%)	254	
	T1067A	rs3894326	Mild	TT	420 (90.4%)	TA	43 (9.64%)	AA	0	463	0.792
			Severe	TT	229 (90.2%)	TA	25 (9.8%)	AA	0	254	

FUT2 and FUT3 allele frequencies and genotypes, and resultant phenotypes were consistent with that known for the Caucasian population. [27] There is no association between lung disease severity in patients homozygous for the ΔF508 mutation and individual FUT2 or FUT 3 SNPs, nor with secretor or Lewis status. ^§^ P-values generated by Fisher exact test.

### FUT3 genotype

Four SNPs account for 90–95% of Lewis status in Caucasians. [23] FUT 3 alleles were successfully determined at these four loci for 707 patients. There was no significant difference for individual SNPs between mild versus severe lung disease patients, nor by gender, meconium ileus or asthma status by Fisher exact test. (See Table 2.) Orntoft et al correlated FUT3 gene mutations with enzyme activity and circulating levels of sialyl-Lewis a [28]; therefore, the SNPs were analyzed in groups consistent with Lewis negative or positive phenotypes, but no significant differences were observed. (Data not shown.) [23]

### ABH Type

We anticipated that the A, B or AB blood group, secretor (FUT2) and Lewis positive (FUT3) genotypes would be found in higher prevalence in patients with severe lung phenotype, because genotypes associated with functional fucosyltransferases for synthesis of pertinent glycoproteins have been shown to be associated with increased propensity towards infection with *P. aeruginosa*, *H. influenzae* and *S. aureus*. [15], [29] Conversely, we thought that O blood group, non-secretor and Lewis negative genotypes (genotypes that lack or produce nonfunctional fucosyltransferases) would have higher prevalence in patients with mild phenotypes, reflecting a reduced risk of infection. In order to evaluate the effect of ABH type (A, B, AB, or O blood type, secretor status, and Lewis status) on risk for severe lung disease, multiple logistic regression was performed using the severity phenotype as a response, and the ABH type variables as predictors. Neither main effects nor interactions of ABH genotypes achieved marginal significance at level alpha = 0.10. (See Supplement Text S1 and Table S4.)

### Acquisition of *P. aeruginosa* and ABH Type

**Figure 1:**
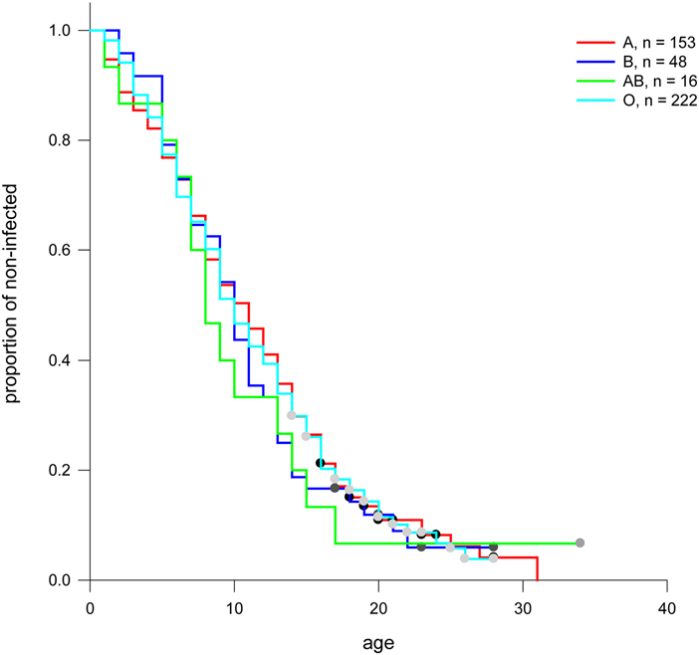
Association between age of onset of persistent *P. aeruginosa* infection and ABH type. Kaplan-Meir curves were generated (includes exit censoring) to test associations between ABH and age of onset of *P. aeruginosa* infection in the respiratory tract for patients with mild and severe disease. (For simplicity, figures show the proportion of infected patients in the combined group of young mild and severe patients for whom culture and ABH genotype data was available.) A) ABO blood group, B) Secretor phenotype and C) Lewis phenotype. No significant differences were seen.

By age 18, up to 80% of CF patients are colonized with *P. aeruginosa.* [7] Thus, in our population of patients, there was no significant difference between *P. aeruginosa* colonization in the mild versus the severe groups at the age of enrollment (mean age 28.6±9.7 years and 16.2±4.1 years, respectively.) In order to determine if ABH status was associated with early infection with *P. aeruginosa,* data from the national CFF Registry was used to determine age of first acquisition of *P. aeruginosa* for each patient (as defined in Methods.) So that the question could be examined in a groups of patients that were similar in age for this comparison, data from both the entire mild group, and from a subset of the mild group (“young milds,” age 15–28 years with a mean age of 20.9±4.0 years) was used. A clear distinction was noted in the age of onset for persistent *P. aeruginosa* infection (age 6.9 years in the severe group, n = 194 versus age12.4 years in the young mild group, n = 224 p<0.0001) [30] Complete data (at least 3 years of *P. aeruginosa* culture data available to define persistent infection, and information for at least one of the three ABH genotypes) was available on 661 patients. Associations between ABO, secretor and Lewis phenotypes and *P. aeruginosa* acquisition were analyzed by Kaplan-Meir plots for patients in the severe group, for patients in the entire mild group, and for patients in the young mild group. No significant differences for ABO blood group, secretor versus non-secretor status, or Lewis positive versus Lewis negative status were observed within groups. Furthermore, no significant differences were seen between patients in the severe group compared to those in the mild group nor in the young mild group based on ABH status. (Figure 1; for simplicity, combined data for patients in the severe and young mild groups is shown. Individual plots for the severe and young mild groups are shown in Supplement Text S1 and Figure S2.)

## Discussion

Although a great deal is known about the pathophysiology of CF lung disease, and more than 1500 CF mutations have been described [17], predictable correlation between CFTR genotype and lung phenotype has not been established. [31] Furthermore, it is known that patients with the same CF gene mutations can have highly variable outcomes. [32] Two studies of CF twins and siblings have shown that most of the disease variation reflects genetic rather than environmental influences [33], [34]; thus, multiple studies are investigating what non-CF genes may play a role in CF phenotype. [20]

We chose to evaluate the ABH genes as candidates for modifiers of CF lung disease severity because the role postulated for these polymorphic genes coding for the ABH antigens is thought to be related to host-pathogen interactions; specifically, many pathogens utilize surface glycoproteins in host invasion. Numerous studies have shown that the binding and infection of humans with microbial organisms is dependent on ABH status; for example, noroviruses (previously Norwalk virus,) certain strains of *S. aureus, H. influenzae, and P. aeruginosa* have this capability. [13], [14] Polymorphisms in the genes coding for the ABH antigens are also known to be associated with various disease states, including pulmonary disease. [11] Patients with COPD who are nonsecretors had significantly lower mean FEV_1_/FVC values than secretors, suggesting that the presence of ABH antigens in respiratory epithelium may be protective in COPD. [17] Similarly, Lewis negative or nonsecretor status was significantly associated with lower lung function, and higher prevalence of wheezing and asthma, in blood group O adults. [18] The association between blood group O/nonsecretor genotype and asthma has also been demonstrated in children. [19], [34] One small study was performed in fifty patients with CF to evaluate whether secretor status was a risk factor for clinical status or *P. aeruginosa* or *S. aureus* colonization. The study found no correlation between secretor status and the clinical variables examined; however, it may have been significantly underpowered to detect a difference with only 17 patients in the nonsecretor group. [35]

Based on previous studies demonstrating an association between ABH genotype and predisposition to infection and lung disease [36], we hypothesized that ABH type might affect lung severity in CF, and genotyped approximately 800 CF patients for ABH type. Although the whole genome scan approach will be used in future genetic modifier studies, the candidate gene approach was chosen to test our hypothesis as only two of the relevant ABH SNPs are included on current SNP-based whole genome platforms.

We examined the ABO, secretor and Lewis status individually in the severe versus mild CF lung disease groups, and found no difference in prevalence of each ABH type between the two groups.

The knowledge that lung disease severity may result from a multitude of other genetic and environmental factors also lead us to hypothesize that more specific phenotypes should be examined for association with ABH genotype, such as female gender, which is associated with worse outcome in CF. [37] Therefore, we evaluated the prevalence of ABH genotypes in males versus females, but no association was found in our CF population.

Although approximately 80% of patients develop chronic Pseudomonal infection by early adulthood, the age of acquisition of *P. aeruginosa* is variable. [8] Based on previous studies demonstrating increased susceptibility to *P. aeruginosa* based on ABH group [16], and increased affinity of *P. aeruginosa* for CF respiratory and salivary mucins [36], [38]–[41], we hypothesized that there would be a difference in prevalence of the ABH antigens amongst those patients with earlier colonization with *P. aeruginosa*. However, there was no difference in prevalence of ABO, secretor or Lewis genotypes associated with age of onset of persistent *P. aeruginosa* whether we looked at all patients, or restricted our analysis to the severe group or to the mild group.

Another phenotype considered was that of meconium ileus (MI). It is known that only 15–20% of CF patients, regardless of CF gene mutation, develop MI. Previous studies of ileal mucus in CF neonates with MI have demonstrated increased fucosylation. [42], [43] CF gene knockout mice, whose main pathology is intestinal mucous obstruction, have increased fucosylation and increased FUT2 expression.[44] In a small study of homo- and heterozygous ΔF508 patients, presence of both functional secretor and Lewis genes was associated with meconium ileus. [45] These studies lead us to hypothesize that MI in CF patients may be related to glycosylation patterns/ABH status; however, no difference in prevalence of ABH genotypes was found between the two groups. Because there are only 129 patients in the MI group, this evaluation may be under powered to detect a difference.

### Limitations

Bacterial colonization and inflammation may appear very early in CF lung disease [34], and prospective cultures were not available; we recognize the limitation of data collected retrospectively for *P. aeruginosa* acquisition and infection. However, we were able to collect culture data on more than 60% of these patients by age 8 years; 73% of the patients with mild disease and 60% of patients with severe disease were culture negative for *P. aeruginosa* at the time of first available culture. Thus, our sample should be adequate to answer the question. However, if ABH genotypes predispose to early bacterial or viral infection, it is possible that we were unable to demonstrate this finding in our group of older children and adults. To definitively determine if there is an effect of differences in ABH genotype on early acquisition of infection and subsequent lung health in CF, it would be necessary to prospectively study this question.

In general, the loss of some patients from an original birth cohort is a limitation to study designs examining variants in severe versus mild forms of the disease. However, according to CF registry data, for the cohort we examined, only 10% have died by age 16 years, thus, there were relatively few deaths in our cohort. Additionally, the patients in our cohort classified as having severe lung disease are still in the worst 25^th^ percentile for their birth cohort, and the patients with mild disease are in the upper 25^th^ percentile of their birth cohort. The validity of these concepts has previously been demonstrated; we reported that *TGFB* genotype is associated with disease severity, and this finding has been replicated in two additional cohorts. [20], [46] Thus, while death of the most severe patients could have potentially influenced the outcome of this study because of the extremes of phenotype design, we do not believe that limitation is a critical factor in the findings reported here.

In conclusion, there is a clear association between the age of onset of chronic *P. aeruginosa* infection and the severity of lung disease, but this association was not found to be related to ABH genotype. We found no association between ABO blood group, or secretor or Lewis status with lung disease severity or *P. aeruginosa* infection status in a large group of CF patients homozygous for the ΔF508 mutation. Nor did we find an association between ABH genotype and gender, asthma or meconium ileus in this patient population. This targeted study has generated important “negative” data regarding modifier genes in CF, which is not possible through SNP-based platforms (e.g. Ilumina 550K chip) because these platforms do not test for all of the pertinent SNPs necessary to establish ABO, Secretor and Lewis status.

## Supporting Information

Text S1 (0.07 MB DOC)

Figure S1 Major pathways of synthesis of the ABH and Lewis antigens (Adapted with permission.) [5] Type I is antigen precursor of ABH antigens in secretions. Le-Lewis positive and le = Lewis negative. See text for more detail. Lewis a/x denotes addition to Type 1 or Type 2 disaccharide precursor. Lewis b/y denotes addition to H antigen derived from Type 1 or Type 2 disaccharide precursor.
(0.13 MB PDF)

Figure S2 Association between age of onset of persistent P. aeruginosa infection and ABH type. Kaplan-Meir curves were generated (includes exit censoring) to test associations between ABH and age of onset of P. aeruginosa infection in the respiratory tract for patients with mild and severe disease. Figures show the proportion of infected patients in the young mild and severe patients for whom culture and ABH genotype data was available. A) ABO blood group in patients with severe disease, B) ABO blood group in young patients with mild disease, C) Secretor phenotype in patients with severe disease D) Secretor phenotype in young patients with mild disease E) Lewis phenotype in patients with severe disease, F) Lewis phenotype in young patients with mild disease. A marginally significant p-value (p = 0.032) was demonstrated in secretor status in patients in the severe group. No other significant differences were seen by Log-Rank test. S-Secretor, NS-Nonsecretor
(3.82 MB TIF)

Table S1 PCR fragments and primers for ABO genotyping. Primers were designed to amplify three fragments from exons 6 and 7 containing the 9 SNPs (rs8176719, rs8176720, rs1053878, rs7853989, rs8176740, rs8176741, rs8176742, rs816750, rs8176472) necessary to identify blood type.
(0.02 MB DOC)

Table S2 Sequence variations in the 7 most common ABO alleles. Base changes resulting in sequence variation. Changes are shown with reference to A1. This research was originally published in (3) © the American Society of Hematology.
(0.03 MB DOC)

Table S3 Reported incidence of ABO, secretor and Lewis phenotypes in the N = 692. The reported incidence of ABO, secretor and Lewis phenotypes is shown in black. [7] Based on our ABH genotyping, the percentage of GMS patients in each category is shown in italics.
(0.03 MB DOC)

Table S4 Multivariate regression analysis. To investigate the effect of blood group alleles (ABO genotype, nonsecretor phenotype, and FUT3 genotypes producing Lewis negative phenotypes) on CF lung severity risk, we performed multiple logistic regression analyses to predict severity status. As previously reported [1], there was a highly statistically significant correlation with the TGF-β codon 10 CC genotype, and a moderately significant association with meconium ileus status. No other variables achieved marginal significance at level alpha = 0.10, including main effects and interactions of ABH genotypes.
(0.04 MB DOC)

## References

[1] Bobadilla JL, Macek M, Fine JP, Farrell PM (2002). Cystic fibrosis: a worldwide analysis of CFTR mutations–correlation with incidence data and application to screening.. Hum Mutat.

[2] Riordan RJ, Kerem BS, Alon N (1989). Identification of the cystic fibrosis gene: cloning and characterization of complementary DNA.. Science.

[3] Cystic Fibrosis Mutation Data Base

[4] Cutting GR (2005). Modifier genetics: cystic fibrosis.. Annu Rev Genomics Hum Genet.

[5] Boyle MP (2007). Strategies for identifying modifier genes in cystic fibrosis.. Proc Am Thorac Soc.

[6] Knowles MR, Boucher RC (2002). Mucus clearance as a primary innate defense mechanism for mammalian airways.. J Clin Invest.

[7] Gibson RL, Burns JL, Ramsey BW (2003). Pathophysiology and management of pulmonary infections in cystic fibrosis.. Am J Respir Crit Care Med.

[8] Li Z, Kosorok MR, Farrell PM, Laxova A, West SE (2005). Longitudinal development of mucoid Pseudomonas aeruginosa infection and lung disease progression in children with cystic fibrosis.. Jama.

[9] Wat D, Gelder C, Hibbitts S, Cafferty F, Bowler I (2008). The role of respiratory viruses in cystic fibrosis.. J Cyst Fibros.

[10] Wang EE, Prober CG, Manson B, Corey M, Levison H (1984). Association of respiratory viral infections with pulmonary deterioration in patients with cystic fibrosis.. N Engl J Med.

[11] Marionneau S, Cailleau-Thomas A, Rocher J, Le Moullac-Vaidye B, Ruvoen N (2001). ABH and Lewis histo-blood group antigens, a model for the meaning of oligosaccharide diversity in the face of a changing world.. Biochimie.

[12] Yip SP (2002). Sequence variation at the human ABO locus.. Ann Hum Genet.

[13] Huang P, Farkas T, Marionneau S, Zhong W, Ruvoen-Clouet N (2003). Noroviruses bind to human ABO, Lewis, and secretor histo-blood group antigens: identification of 4 distinct strain-specific patterns.. J Infect Dis.

[14] Marionneau S, Airaud F, Bovin NV, Le Pendu J, Ruvoen-Clouet N (2005). Influence of the combined ABO, FUT2, and FUT3 polymorphism on susceptibility to Norwalk virus attachment.. J Infect Dis.

[15] Saadi AT, Weir DM, Poxton IR, Stewart J, Essery SD (1994). Isolation of an adhesin from Staphylococcus aureus that binds Lewis a blood group antigen and its relevance to sudden infant death syndrome.. FEMS Immunol Med Microbiol.

[16] Wu AM, Wu JH, Singh T, Liu JH, Tsai MS (2006). Interactions of the fucose-specific Pseudomonas aeruginosa lectin, PA-IIL, with mammalian glycoconjugates bearing polyvalent Lewis(a) and ABH blood group glycotopes.. Biochimie.

[17] Cohen BH, Bias WB, Chase GA, Diamond EL, Graves CG (1980). Is ABH nonsecretor status a risk factor for obstructive lung disease?. Am J Epidemiol.

[18] Kauffmann F, Frette C, Pham QT, Nafissi S, Bertrand JP (1996). Associations of blood group-related antigens to FEV1, wheezing, and asthma.. Am J Respir Crit Care Med.

[19] Ronchetti F, Villa MP, Ronchetti R, Bonci E, Latini L (2001). ABO/Secretor genetic complex and susceptibility to asthma in childhood.. Eur Respir J.

[20] Drumm ML, Konstan MW, Schluchter MD, Handler A, Pace R (2005). Genetic modifiers of lung disease in cystic fibrosis.. N Engl J Med.

[21] Schluchter MD, Konstan MW, Drumm ML, Yankaskas JR, Knowles MR (2006). Classifying severity of cystic fibrosis lung disease using longitudinal pulmonary function data.. Am J Respir Crit Care Med.

[22] Svensson L, Petersson A, Henry SM (2000). Secretor genotyping for A385T, G428A, C571T, C628T, 685delTGG, G849A, and other mutations from a single PCR.. Transfusion.

[23] Salomaa V, Pankow J, Heiss G, Cakir B, Eckfeldt JH (2000). Genetic background of Lewis negative blood group phenotype and its association with atherosclerotic disease in the NHLBI family heart study.. J Intern Med.

[24] Cameron HS, Szczepaniak D, Weston BW (1995). Expression of human chromosome 19p alpha(1,3)-fucosyltransferase genes in normal tissues. Alternative splicing, polyadenylation, and isoforms.. J Biol Chem.

[25] Pang H, Liu Y, Koda Y, Soejima M, Jia J (1998). Five novel missense mutations of the Lewis gene (FUT3) in African (Xhosa) and Caucasian populations in South Africa.. Hum Genet.

[26] Harmening D, Harmening D (1999). Modern Blood Banking and Transfusion Practice;.

[27] dbSNP

[28] Orntoft TF, Vestergaard EM, Holmes E, Jakobsen JS, Grunnet N (1996). Influence of Lewis alpha1-3/4-L-fucosyltransferase (FUT3) gene mutations on enzyme activity, erythrocyte phenotyping, and circulating tumor marker sialyl-Lewis a levels.. J Biol Chem.

[29] Rhim AD, Stoykova L, Glick MC, Scanlin TF (2001). Terminal glycosylation in cystic fibrosis (CF): a review emphasizing the airway epithelial cell.. Glycoconj J.

[30] Pittman J CH, Yeatts J, Drumm M, Leigh M, Davis S, Van Rie A, Emomnd M, Knowles M (2008). Age of Pseudomonas Aeruginosa Infection and Severity of Adult Lung Disease in Cystic Fibrosis.. Pediatric Pulmonology.

[31] Rowntree RK, Harris A (2003). The phenotypic consequences of CFTR mutations.. Ann Hum Genet.

[32] Kerem E, Corey M, Kerem BS, Rommens J, Markiewicz D (1990). The relation between genotype and phenotype in cystic fibrosis–analysis of the most common mutation (delta F508).. N Engl J Med.

[33] Vanscoy LL, Blackman SM, Collaco JM, Bowers A, Lai T (2007). Heritability of lung disease severity in cystic fibrosis.. Am J Respir Crit Care Med.

[34] Khan TZ, Wagener JS, Bost T, Martinez J, Accurso FJ (1995). Early pulmonary inflammation in infants with cystic fibrosis.. Am J Respir Crit Care Med.

[35] Haponik EF, Stokes D, Rosenstein BJ, Hughes WT (1985). ABH secretor status in cystic fibrosis–a negative report.. Eur J Respir Dis.

[36] Shori DK, Genter T, Hansen J, Koch C, Wyatt H (2001). Altered sialyl- and fucosyl-linkage on mucins in cystic fibrosis patients promotes formation of the sialyl-Lewis X determinant on salivary MUC-5B and MUC-7.. Pflugers Arch.

[37] Zemel BS, Jawad AF, FitzSimmons S, Stallings VA (2000). Longitudinal relationship among growth, nutritional status, and pulmonary function in children with cystic fibrosis: analysis of the Cystic Fibrosis Foundation National CF Patient Registry.. J Pediatr.

[38] Imundo L, Barasch J, Prince A, Al-Awqati Q (1995). Cystic fibrosis epithelial cells have a receptor for pathogenic bacteria on their apical surface.. Proc Natl Acad Sci U S A.

[39] Devaraj N, Sheykhnazari M, Warren WS, Bhavanandan VP (1994). Differential binding of Pseudomonas aeruginosa to normal and cystic fibrosis tracheobronchial mucins.. Glycobiology.

[40] Carnoy C, Ramphal R, Scharfman A, Lo-Guidice JM, Houdret N (1993). Altered carbohydrate composition of salivary mucins from patients with cystic fibrosis and the adhesion of Pseudomonas aeruginosa.. Am J Respir Cell Mol Biol.

[41] Komiyama K, Habbick BF, Tumber SK (1987). Role of sialic acid in saliva-mediated aggregation of Pseudomonas aeruginosa isolated from cystic fibrosis patients.. Infect Immun.

[42] Clamp JR, Gough M (1979). Study of the oligosaccharide units from mucus glycoproteins of meconium from normal infants and from cases of cystic fibrosis with meconium ileus.. Clin Sci (Lond).

[43] Thiru S, Devereux G, King A (1990). Abnormal fucosylation of ileal mucus in cystic fibrosis: I. A histochemical study using peroxidase labelled lectins.. J Clin Pathol.

[44] Thomsson KA, Hinojosa-Kurtzberg M, Axelsson KA, Domino SE, Lowe JB (2002). Intestinal mucins from cystic fibrosis mice show increased fucosylation due to an induced Fucalpha1-2 glycosyltransferase.. Biochem J.

[45] Elmgren A, Grahn A, Gronowitz E, Aberg L, Heikenheimo M (2002). Lewis and secretor genotypes modify the phenotype in patients with cystic fibrosis.. Pediatric Pulmonology.

[46] Bremer LA, Blackman SM, Vanscoy LL, McDougal KE, Bowers A (2008). Interaction between a novel TGFB1 haplotype and CFTR genotype is associated with improved lung function in cystic fibrosis.. Hum Mol Genet.

