# Supplementary material for: Histo-Blood Group Gene Polymorphisms as Potential Genetic Modifiers of Infection and Cystic Fibrosis Lung Disease Severity
Source: PLoS One. 2009 Jan 26;4(1):e4270. doi: 10.1371/journal.pone.0004270 (PMC2627933; doi:10.1371/journal.pone.0004270)
Supplement: Text S1 — (0.07 MB DOC) [file pone.0004270.s001.doc]

**Histo-blood Group Gene Polymorphisms as Potential Genetic Modifiers of Infection and Cystic Fibrosis Lung Disease Severity**

**Jennifer L Taylor-Cousar^1^*, Maimoona A Zariwala^2^, Lauranell H Burch^3^, Rhonda G Pace^2^, Mitchell L Drumm^6^, Hollin Calloway^2^, Haiying Fan^5^, Brent W Weston^5^, Fred A Wright^4^, Michael R Knowles^2^**^*^

**^1^University of New Mexico Health Sciences Center, Pulmonary Divisions, Internal Medicine and Pediatrics, Albuquerque, NM, USA. ^2^University of North Carolina, Cystic Fibrosis/Pulmonary Research and Treatment Center, Chapel Hill, NC, USA. ^3^National Institute of Environmental Health Sciences, Research Triangle Park, NC, USA. ^4^University of North Carolina, Department of Biostatistics, Chapel Hill, NC, USA. ^5^University of North Carolina, Hematology-Oncology, Department of Pediatrics, Chapel Hill, NC, USA. ^6^Case Western Reserve University, Pediatric Pulmonary Division, Department of Pediatrics, Cleveland, OH, USA**

*Reporting for the Gene Modifier Study Group

#### Supplement

#### Methods (supplement)

Participants

Patients with Cystic Fibrosis who are homozygous for the ΔF508 CF gene mutation were enrolled from 44 sites (n=808 patients) as previously published. [1] Patients were enrolled based on FEV_1_ values in the lowest quartile (“severe;” n=263) or highest quartile (“mild;” n=545) for age. FEV_1_ data has been analyzed according to biostatistical models based on longitudinal lung function (FEV_1._) This modeling has robust power to predict survival; hence the use of multiple spirometric values is a strong surrogate for long-term outcomes. [2]

ABO Genotype

Sequence variation in 7 ABO alleles accounts for >90% of the variants in white Europeans. [3] Primers were designed to amplify three fragments from exons 6 and 7 containing the 9 SNPs (rs8176719, rs8176720, rs1053878, rs7853989, rs8176740, rs8176741, rs8176742, rs816750, rs8176472) necessary to identify blood type. (Table S1) PCR was performed using standard techniques, and high throughput sequencing was performed on an ABI Prism® 3730 Genetic Analyzer. Batch data analysis was performed with SNP finding via the Polyphred© program. In order to determine ABO phenotype (A, B, AB, or O blood group) for each subject, alleles were determined based on known sequence variation (Table S2) and blood type recorded.

FUT2 Genotype

In Caucasians, one nonsynonymous SNP in FUT2 (G428A, rs601338) accounts for >95% of the incidence of nonsecretor phenotype. [4] Allele identification was performed using an ABI Taqman© assay on an ABI Prism® 7700HT/7900HT Sequence Detection System. Loss of function mutations in FUT2 (non-secretor) occur in 20% of European-derived Caucasians. [5] Genotypes G/G and G/A are consistent with the secretor phenotype, and genotype AA is consistent with the non-secretor phenotype.

FUT3 Genotype

SNPs in four positions (T59G, rs28362459; T202C, rs812936; C314T, rs778986; and T1067A, rs3894326) account for 90-95 % of Lewis status in Caucasians.[5] Primers were designed for PCR amplification and sequencing of the FUT3 gene was performed on an ABI Prism® 3130xl/310 Genetic Analyzer. Loss of function mutations in FUT3 (Lewis negative) occur in 10% of European-derived Caucasians. [5] Orntoft et al correlated FUT3 gene mutations with enzyme activity and circulating levels of sialyl-Lewis a, whereby healthy heterozygotes had significantly less α-1, 4 fucosyltransferase activity in saliva than did wild-type homozygotes, and lower enzyme activity was associated with significantly lower circulating levels of sialyl-Lewis a. [6] Therefore, in addition to analysis of individual FUT3 SNPs, the SNPs were analyzed in groups consistent with Lewis negative or positive phenotypes.

Acquisition of *P. aeruginosa* and ABH Type

In order to determine if ABH status was associated with early colonization with *P. aeruginosa,* data from the national CFF Registry was used to determine age of first acquisition of *P. aeruginosa* for each patient. Age of acquisition was defined as the first age at which *P. aeruginosa* was present in respiratory cultures in two consecutive, or two out of three years. So that the question could be examined in groups of patients that were similar in age for this comparison, data from both the entire mild group, and from a subset of the mild group (“young milds,” age 15-28 years with a mean age of 20.9±4.0 years) was utilized.

Statistical Analysis

To investigate the effect of blood group alleles (ABO genotype, nonsecretor phenotype, and

FUT3 genotypes producing Lewis negative phenotypes) on CF lung severity risk, we performed

multiple logistic regression analyses to predict severity status. The number of A and B antigen

alleles was included in an additive model on the log-odds of severe lung status, as well as the

FUT2 and FUT3 genotypes, and terms to account for possible interaction between the genes.  In

addition, the following predictors were used in the model: gender, meconium ileus, asthma, and

the TGFb1 codon 10 CC genotype (previously shown to be associated with severe lung disease.)

**Results**

Characteristics of the patients

As previously reported, pulmonary and nutrition characteristics of the patients classified as severe (n=263) were markedly different than that of patients classified as mild (n=545.) [1] Although the mean age of the patients in the mild group was 28.6 ± 9.7 years compared to 16.2 ± 4.2 years in the severe group, FEV_1_ was more than 25% higher and body mass index percentile was 24% higher in the patients in the mild group versus those in the severe group (p<0.001). In order to have a comparison group that was similar in age in to the severe group, the mild cohort was divided into two groups, over 28 years of age, and under 28 years of age. These two groups were validated to be congruent by concordance analysis. [1] The young milds are a group of patients who ranged in age from 15-28 years, and whose mean age was 20.9±4.0 years. Because of the association between age and acquisition of *P. aeruginosa*, in addition to comparing acquisition of *P. aeruginosa* in patients in the mild and severe groups, we sought to compare groups that were more similar in age. Thus, age of acquisition of *P. aeruginosa* was also compared in the young milds. No results were found to be significant.

ABH Genotype

To insure accuracy of our genotyping for ABO, FUT2 and FUT3 alleles, data obtained in this

study was compared to what is reported for ABH phenotypes in the Caucasian population.

ABO blood group, FUT2 (secretor positive or negative status), and FUT3 (Lewis

positive or negative status) in our patient population were consistent with that reported for

Caucasians. [7,8] (Table S3)

Acquisition of *P. aeruginosa* and ABH Type

A clear distinction was noted in the age of onset for persistent *P. aeruginosa* infection (age 6.9 years in the severe group, n=194 versus age12.4 years in the young mild group, n=224 p<0.0001) [9] Complete data (at least 3 years of *P. aeruginosa* culture data available to define persistent infection*,* and information for at least one of the three ABH genotypes) was available on 661 patients. Associations between ABO, secretor and Lewis phenotypes and *P. aeruginosa* acquisition were analyzed by Kaplan-Meir plots for patients in the severe group, for patients in the entire mild group, and for patients in the young mild group. A marginally significant p-value (p=0.032) was observed between secretor negative and secretor positive patients in the severe group. However, no other significant differences for ABO blood group, secretor versus non-secretor status nor Lewis positive versus Lewis negative status were observed within groups. Furthermore, no significant differences were seen between patients in the severe group compared to those in the mild group, nor in the young mild group based on ABH status. (Figure S2, data shown for severes and young milds.)

ABH Type

In order to evaluate the effect of ABH type (A, B, AB, or O blood type, secretor status, and Lewis status) on risk for severe lung disease, multiple logistic regression was performed, using the severity phenotype as a response and the ABH type variables as predictors. Additional covariates were included that have been previously shown to influence severity, including gender, meconium ileus, asthma, and the TGF ß codon 10 CC genotype. As previously reported [1], there was a highly statistically significant correlation with the TGF-β codon 10 CC genotype, and a moderately significant association with meconium ileus status. No other variables achieved marginal significance at level alpha=0.10, including main effects and interactions of ABH genotypes. (Table S4)

**References (supplement)**

1. Drumm ML, Konstan MW, Schluchter MD, Handler A, Pace R, et al. (2005) Genetic modifiers of lung disease in cystic fibrosis. N Engl J Med 353: 1443-1453.

2. Schluchter MD, Konstan MW, Drumm ML, Yankaskas JR, Knowles MR (2006) Classifying severity of cystic fibrosis lung disease using longitudinal pulmonary function data. Am J Respir Crit Care Med 174: 780-786.

3. Yip SP (2002) Sequence variation at the human ABO locus. Ann Hum Genet 66: 1-27.

4. Svensson L, Petersson A, Henry SM (2000) Secretor genotyping for A385T, G428A, C571T, C628T, 685delTGG, G849A, and other mutations from a single PCR. Transfusion 40: 856-860.

5. Salomaa V, Pankow J, Heiss G, Cakir B, Eckfeldt JH, et al. (2000) Genetic background of Lewis negative blood group phenotype and its association with atherosclerotic disease in the NHLBI family heart study. J Intern Med 247: 689-698.

6. Orntoft TF, Vestergaard EM, Holmes E, Jakobsen JS, Grunnet N, et al. (1996) Influence of Lewis alpha1-3/4-L-fucosyltransferase (FUT3) gene mutations on enzyme activity, erythrocyte phenotyping, and circulating tumor marker sialyl-Lewis a levels. J Biol Chem 271: 32260-32268.

7. Harmening D (1999) Modern Blood Banking and Transfusion Practice; Harmening D, editor. Philadelphia: F.A. Davis Company.

8. dbSNP.

9. Pittman J CH, Yeatts J, Drumm M, Leigh M, Davis S, Van Rie A, Emomnd M, Knowles M (2008) Age of Pseudomonas Aeruginosa Infection and Severity of Adult Lung Disease in Cystic Fibrosis. Pediatric Pulmonology 43: A332.

**Figure Legends**

**Figure S1. Major pathways of synthesis of the ABH and Lewis antigens** (Adapted with permission.) [5]  **Type I** is antigen precursor of ABH antigens in secretions. **Le**-Lewis positive and **le**=Lewis negative. See text for more detail. Lewis a/x denotes addition to Type 1 or Type 2 disaccharide precursor. Lewis b/y denotes addition to H antigen derived from Type 1 or Type 2 disaccharide precursor.

**Figure S2**. **Association between age of onset of persistent *P. aeruginosa* infection and ABH type.** Kaplan-Meir curves were generated (includes exit censoring) to test associations between ABH and age of onset of *P. aeruginosa* infection in the respiratory tract for patients with mild and severe disease. Figures show the proportion of infected patients in the young mild and severe patients for whom culture and ABH genotype data was available. A) ABO blood group in patients with severe disease, B) ABO blood group in young patients with mild disease, C) Secretor phenotype in patients with severe disease D) Secretor phenotype in young patients with mild disease E) Lewis phenotype in patients with severe disease, F) Lewis phenotype in young patients with mild disease. A marginally significant p-value (p=0.032) was demonstrated in secretor status in patients in the severe group. No other significant differences were seen by Log-Rank test. S-Secretor, NS-Nonsecretor
