# Supplementary figures and images for: Histo-Blood Group Gene Polymorphisms as Potential Genetic Modifiers of Infection and Cystic Fibrosis Lung Disease Severity
Source: PLoS One. 2009 Jan 26;4(1):e4270. doi: 10.1371/journal.pone.0004270 (PMC2627933; doi:10.1371/journal.pone.0004270)

**Figure S1.**

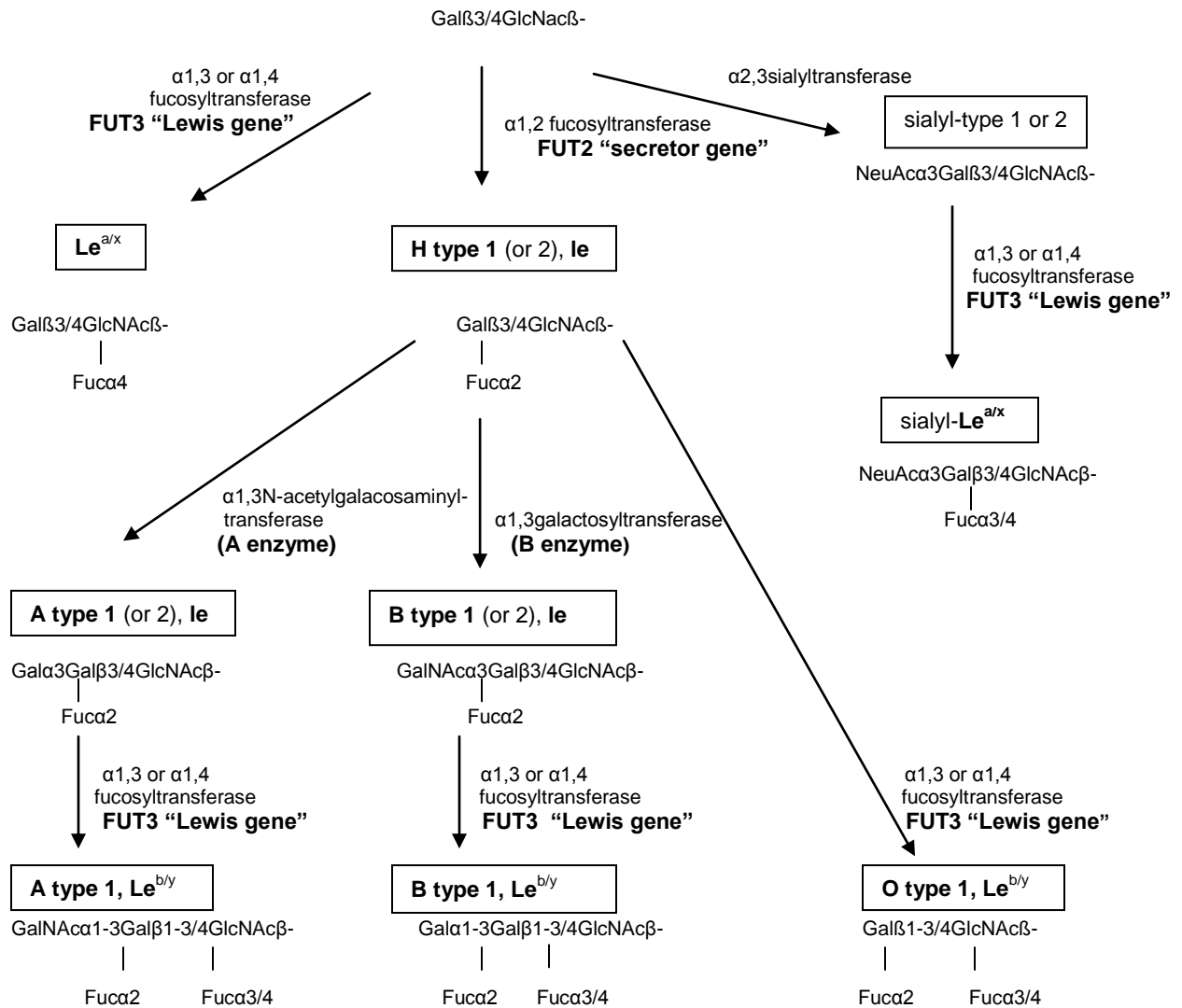

Supplement: Figure S1 — Major pathways of synthesis of the ABH and Lewis antigens (Adapted with permission.) [5] Type I is antigen precursor of ABH antigens in secretions. Le-Lewis positive and le = Lewis negative. See text for more detail. Lewis a/x denotes addition to Type 1 or Type 2 disaccharide precursor. Lewis b/y denotes addition to H antigen derived from Type 1 or Type 2 disaccharide precursor. (0.13 MB PDF) [file pone.0004270.s002.pdf]

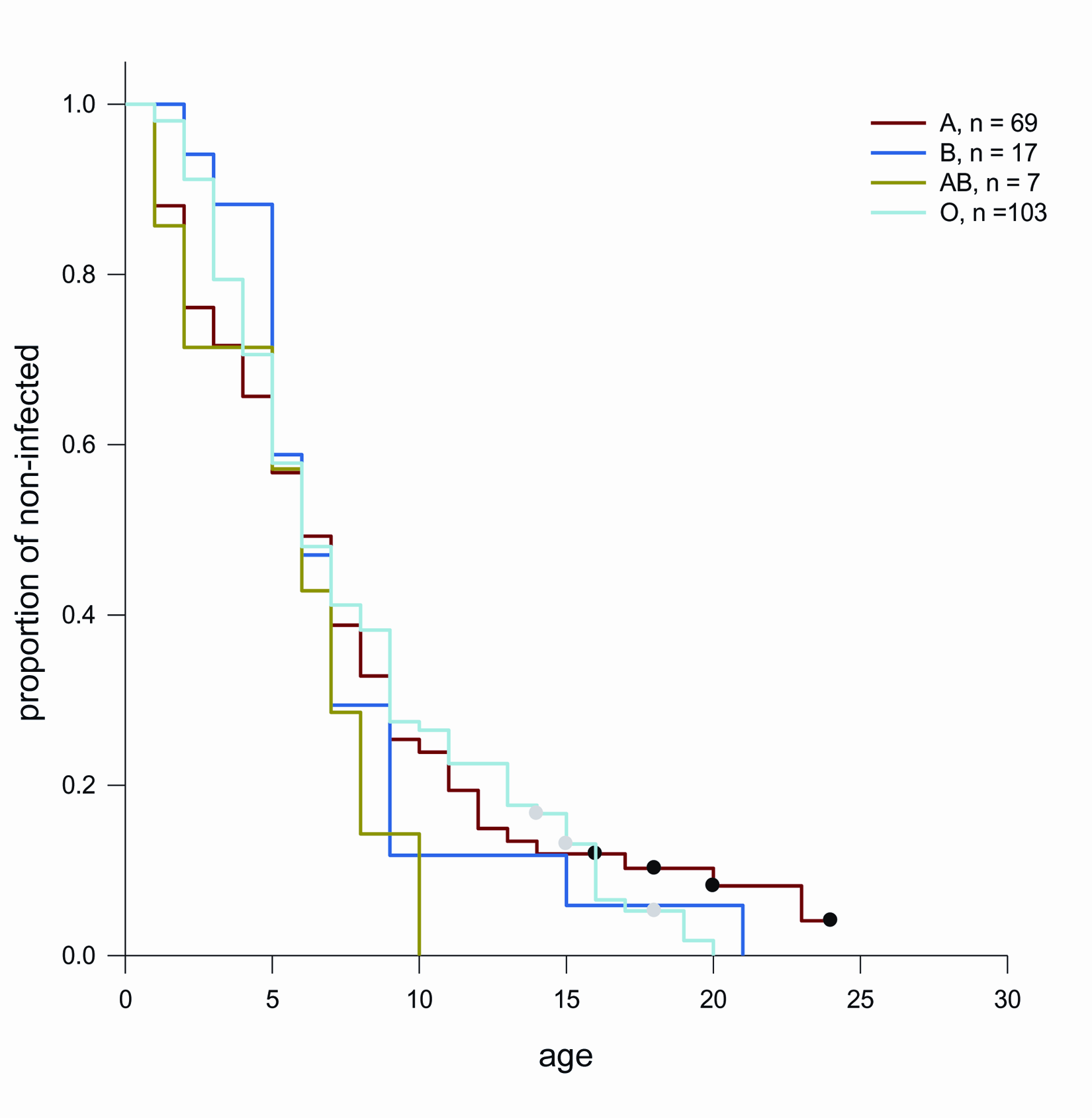

Supplement: Figure S2 — Association between age of onset of persistent P. aeruginosa infection and ABH type. Kaplan-Meir curves were generated (includes exit censoring) to test associations between ABH and age of onset of P. aeruginosa infection in the respiratory tract for patients with mild and severe disease. Figures show the proportion of infected patients in the young mild and severe patients for whom culture and ABH genotype data was available. A) ABO blood group in patients with severe disease, B) ABO blood group in young patients with mild disease, C) Secretor phenotype in patients with severe disease D) Secretor phenotype in young patients with mild disease E) Lewis phenotype in patients with severe disease, F) Lewis phenotype in young patients with mild disease. A marginally significant p-value (p = 0.032) was demonstrated in secretor status in patients in the severe group. No other significant differences were seen by Log-Rank test. S-Secretor, NS-Nonsecretor (3.82 MB TIF) [file pone.0004270.s003.tif]
