# Supplementary material for: Histo-Blood Group Gene Polymorphisms as Potential Genetic Modifiers of Infection and Cystic Fibrosis Lung Disease Severity
Source: PLoS One. 2009 Jan 26;4(1):e4270. doi: 10.1371/journal.pone.0004270 (PMC2627933; doi:10.1371/journal.pone.0004270)
Supplement: Table S1 — PCR fragments and primers for ABO genotyping. Primers were designed to amplify three fragments from exons 6 and 7 containing the 9 SNPs (rs8176719, rs8176720, rs1053878, rs7853989, rs8176740, rs8176741, rs8176742, rs816750, rs8176472) necessary to identify blood type. (0.02 MB DOC) [file pone.0004270.s004.doc]

Primer Name Sequence (5’-3’)

ABOex6seqF GAGGCAGAAGCTGAGTGGAG

ABOex6seqR CTGCATGAATGACCTTTCCC

ABOex7PCRf CATCTGCTGCTCTAAGCCTTCC

ABOex7seqf1 TGCAGATACGTGGCTTTCCTG

ABOex7seqr1 CGCTCGTAGGTGAAGGCCTC

ABOex7seqf2 GAGGCCTTCACCTACGAGCG

ABOex7seqr2 CAAAGGAAACAGAGTTTACCCGTTC

ABOex7PCRr GCCTAGGCTTCAGTTACTCACAACAG

**Table S1. PCR fragments and primers for ABO genotyping.** Primers were designed to amplify three fragments from exons 6 and 7 containing the 9 SNPs (rs8176719, rs8176720, rs1053878, rs7853989, rs8176740, rs8176741, rs8176742, rs816750, rs8176472) necessary to identify blood type.
