# Supplementary material for: Histo-Blood Group Gene Polymorphisms as Potential Genetic Modifiers of Infection and Cystic Fibrosis Lung Disease Severity
Source: PLoS One. 2009 Jan 26;4(1):e4270. doi: 10.1371/journal.pone.0004270 (PMC2627933; doi:10.1371/journal.pone.0004270)
Supplement: Table S2 — Sequence variations in the 7 most common ABO alleles. Base changes resulting in sequence variation. Changes are shown with reference to A1. This research was originally published in (3) © the American Society of Hematology. (0.03 MB DOC) [file pone.0004270.s005.doc]

|  | **EXON** | **6** |  | **7** |  |  |  |  |  |
| --- | --- | --- | --- | --- | --- | --- | --- | --- | --- |
|  |  |  |  |  |  |  |  |  |  |
| **NUCLEOTIDE** | 261 | 297 | 467 | 526 | 646 | 657 | 681 | 1059-61 | 1096 |
| A^1^ | G | A | C | C | T | C | G | C | G |
| A^1^v |  |  | T |  |  |  |  |  |  |
| A^2^ |  |  | T |  |  |  |  |  |  |
| B |  | G |  | G |  | T |  |  | A |
| O^1^ | --- |  |  |  |  |  |  |  |  |
| O^1^v | --- | G |  |  | A |  | A |  |  |
| O^2^ |  | G |  | G |  |  |  |  | A |

**Table S2**. **Sequence variations in the 7 most common ABO alleles.** Base changes resulting in sequence variation. Changes are shown with reference to A^1^. This research was originally

published in (3) © the American Society of Hematology.
