# Supplementary material for: Histo-Blood Group Gene Polymorphisms as Potential Genetic Modifiers of Infection and Cystic Fibrosis Lung Disease Severity
Source: PLoS One. 2009 Jan 26;4(1):e4270. doi: 10.1371/journal.pone.0004270 (PMC2627933; doi:10.1371/journal.pone.0004270)
Supplement: Table S3 — Reported incidence of ABO, secretor and Lewis phenotypes in the N = 692. The reported incidence of ABO, secretor and Lewis phenotypes is shown in black. [7] Based on our ABH genotyping, the percentage of GMS patients in each category is shown in italics. (0.03 MB DOC) [file pone.0004270.s006.doc]

|  | **Incidence of**  **FUT2/FUT3** | **seLe 22%** | **SeLe 72%** | **sele 1.2%** | **Sele 4.8%** |
| --- | --- | --- | --- | --- | --- |
|  |  | *(22.1%)* | *(69.4%)* | *(2.3%)* | *(6.2%)* |
| **Incidence of ABO**  **type** |  |  |  |  |  |
| **45% O** |  | 9.9% | 32% | 0.5% | 2% |
| ***(****47.8%)* |  | *(11.0%)* | *(32.5%)* | *(1.3%)* | *(3.0%)* |
| **10% B** |  | 2.2% | 7.2% | 0.1% | 0.5% |
| *(10.3%)* |  | *(2.2%)* | *(7.4%)* | *(0.1%)* | *(0.6%)* |
| **41% A** |  | 9% | 30% | 0.5% | 2% |
| *(38.1%)* |  | *(8.5%)* | *(26.3%)* | *(0.6%)* | *(2.6%)* |
| **4% AB** |  | 0.9% | 3% | 0.05% | 0.2% |
| *(3.7%)* |  | *(0.4%)* | *(3.2%)* | *(0.1%)* | *(0%)* |

**Table S3**. **Reported incidence of ABO, secretor and Lewis phenotypes in the**

**Caucasian population and demonstrated incidence in GMS study patients.**

N=692 The reported incidence of ABO, secretor and Lewis phenotypes is shown in black.

[7] Based on our ABH genotyping, the percentage of GMS patients in each category is

shown in italics.
