# Supplementary material for: Histo-Blood Group Gene Polymorphisms as Potential Genetic Modifiers of Infection and Cystic Fibrosis Lung Disease Severity
Source: PLoS One. 2009 Jan 26;4(1):e4270. doi: 10.1371/journal.pone.0004270 (PMC2627933; doi:10.1371/journal.pone.0004270)
Supplement: Table S4 — Multivariate regression analysis. To investigate the effect of blood group alleles (ABO genotype, nonsecretor phenotype, and FUT3 genotypes producing Lewis negative phenotypes) on CF lung severity risk, we performed multiple logistic regression analyses to predict severity status. As previously reported [1], there was a highly statistically significant correlation with the TGF-β codon 10 CC genotype, and a moderately significant association with meconium ileus status. No other variables achieved marginal significance at level alpha = 0.10, including main effects and interactions of ABH genotypes. (0.04 MB DOC) [file pone.0004270.s007.doc]

**Logistic regression of lung severity phenotype on clinical and genetic predictors*.**

| **Variable** | **Coefficient** | **Std. Error** | **z value** | **p-value** |
| --- | --- | --- | --- | --- |
| Intercept | -0.8893 | 0.3692 | -2.409 | **0.0160** |
| Gender (1=male,2=female) | 0.2242 | 0.1649 | 1.360 | 0.1740 |
| MI status** | 0.5542 | 0.2151 | 2.576 | **0.0100** |
| Asthma status | -0.3568 | 0.2105 | -1.695 | 0.0900 |
| Codon 10 CC genotype status | 0.9425 | 0.2339 | 4.029 | **0.000056** |
| ABH no. of A alleles | 0.1923 | 0.3438 | 0.559 | 0.5760 |
| ABH no. of B alleles | -1.1429 | 0.7137 | -1.601 | 0.1093 |
| FUT2 AA genotype status | -0.2390 | 0.2621 | -0.912 | 0.3618 |
| FUT3 (0=N, 1=S, 2=P) | -0.1244 | 0.3440 | -0.361 | 0.7177 |
| (ABH no. of A alleles) X FUT2 AA genotype status | 0.1051 | 0.3087 | 0.340 | 0.7336 |
| (ABH no. of B alleles) X FUT2 AA genotype status | 0.5665 | 0.5926 | 0.956 | 0.3391 |
| (ABH no. of A alleles) X FUT3 | -0.3288 | 0.4150 | -0.792 | 0.4281 |
| (ABH no. of B alleles) X FUT3 | 0.8697 | 0.8508 | 1.022 | 0.3067 |

*Regression model *p*= 0.001. *p*-values in bold are <0.05. For the regression model with the significant predictors MI status and Codon 10 CC genotype removed, model *p*= 0.35

**Each variable labeled “status” is an indicator variable, coded 1 for presence, 0 for absence.

**Table S4. Multivariate regression analysis**. To investigate the effect of blood group alleles

(ABO genotype, nonsecretor phenotype, and FUT3 genotypes producing Lewis negative

phenotypes) on CF lung severity risk, we performed multiple logistic regression analyses to

predict severity status. As previously reported [1], there was a highly statistically significant

correlation with the TGF-β codon 10 CC genotype, and a moderately significant association with

meconium ileus status. No other variables achieved marginal significance at level alpha=0.10,

including main effects and interactions of ABH genotypes.
